# Public Health Challenges for Post-secondary Students During COVID-19: A Scoping Review

**DOI:** 10.1177/2752535X241257561

**Published:** 2024-05-31

**Authors:** Pooja Dey, Leanne R. De Souza

**Affiliations:** 1 Dalla Lana School of Public Health, 98586 University of Toronto, Toronto, ON, Canada; 2 7938 University College, University of Toronto, Toronto, ON, Canada; 3 Factor-Inwentash Faculty of Social Work, Institute for Life Course and Aging, University of Toronto,Toronto, ON, Canada

**Keywords:** undergraduate, health equity, COVID-19, post-secondary, well-being, social determinants of health

## Abstract

Research about public health impacts of COVID-19 on post-secondary students is slowly beginning to emerge. This scoping review identified common public health challenges among post-secondary students in higher-income countries during the COVID-19 pandemic. Five databases were searched to find relevant peer-reviewed literature up to March 2022. Results were categorized according to reported public health challenges and relevant socio-economic variables. After screening, 53 articles were reviewed. Most articles were from the USA (39/53). The seven main public health challenges identified were mental health (35/53), financial instability (25/53), physical health (13/53), food insecurity (12/53), social well-being (8/53), digital access (7/53), and housing or relocation (6/53). Students with low socioeconomic status experienced heightened public health challenges. This review offers insight and opportunities for the development of longitudinal tools to support social determinants of health in post-secondary populations in high-income countries and may offer insight into similar experiences for students in other settings.

## Introduction

The COVID-19 pandemic has caused unprecedented global challenges, that include a significant impact on the higher education sector. With the closure of educational institutions to control transmission rates, post-secondary students struggled at the intersection of access to new modalities for learning, mental and physical health concerns, and a rapidly changing global economy.^1^ Moreover, the emergency transition from in-person instruction to online learning has reportedly resulted in significant barriers for post-secondary students in both low-income and high-income countries, extending beyond impacts on academic performance into physical and mental health challenges.^1^ The unique needs of post-secondary students adapting to the evolving pandemic/post-pandemic can generate new and exacerbate existing public health challenges. Education is an important social determinant of health that supports the building of social capital and upward mobility.^2^ Indeed, the impact of COVID-19 on the global economy has a ripple effect of negative outcomes that are apparent across all the social determinants of health.^3^ While research on post-secondary education in low-income countries often focuses on improving access, analogous research in high-income countries tends to focus on reform.^4^ Both approaches have the potential to address inequities within nations. However, despite the high-income status of a country, inequities are often neglected, especially for low-socioeconomic groups, and require more attention.

Post-secondary education can range from bachelor’s degrees to graduate degrees, associate degrees in technical/community colleges, as well as continuing education, which can demonstrate the diversity of challenges associated with this population. Studies exploring barriers to learning prior to the COVID-19 pandemic reported food,^5^ financial, and housing insecurity,^6^ and digital inequality.^7^ The period during and after COVID-19 - which includes emergency teaching and the adaptation to online and hybrid learning - will mark a significant turning point in the course of post-secondary education, with potential pandemic-specific impacts on student health and well-being.^8^ The degree to which students experience these detrimental impacts may be exacerbated by pre-existing underlying socioeconomic inequities.^9^

In the aftermath of the COVID-19 pandemic, there are clear impacts on post-secondary students that might be effectively addressed by developing equitable policies that relate to online learning and are critical to student health and well-being.^2^ In this study, we conducted an exploratory scoping review to provide a synthesis of public health-related challenges experienced by post-secondary students in OECD (Organization for Economic Cooperation and Development) ‘high-income’ countries during the COVID-19 pandemic. In this study, OECD refers to a membership of high-income countries that have similar social, economic, and educational systems. To our knowledge, this is the first scoping review that synthesizes the evidence on the public health impacts of emergency online learning during the COVID-19 pandemic on post-secondary student health and well-being.

## Methods

This scoping review followed the Arksey and O’Malley methodological framework.^10^ Given the exploratory nature of this research, a scoping review is appropriate to “map the key concepts underpinning a research area as well as to clarify working definitions”.^10,11^ The six stages of the scoping review include: (1) Identifying the research question, (2) Identifying relevant studies, (3) Study selection, (4) Charting data, (5) Collating, summarizing and reporting the results, and (6) Consultation exercise.^10^ Due to resource and time constraints, the consultation exercise was not performed.

### Stage 1: Identifying the Research Question

The focus of this review was to explore the various public health challenges that post-secondary students encountered during the COVID-19 pandemic and the health implications for this vulnerable population. Therefore, the following research questions guided the literature search:

1. What are the public health experiences and challenges that post-secondary students in OECD (Organization for Economic Cooperation and Development) countries encountered during the COVID-19 pandemic (2020-2022)?
2. What are the potential socioeconomic disparities underlying the public health challenges experienced by post-secondary students in OECD countries during the COVID-19 pandemic?

### Stage 2: Identifying Relevant Studies

A broad selection of keywords and subject headings, adapted for each database, were used to guide our search strategy. Databases searched included OVID Medline, EBSCO Education Source, ERIC, Scopus, and PsycInfo to identify relevant peer-reviewed literature. Key search terms included “post-secondary”, “COVID-19”, “online learning”, “inequalit*”, “financial stress”, “food insecurity” and “digital inequality*”. Two search rounds were conducted, where the last search round before study completion accounted for new additions to the literature. A detailed search strategy for each database is outlined in the Supplemental Materials. Consultation with a university librarian ensured the refinement of key terms and database choices. Grey literature was not searched as most reports were anticipated to come from associations that cite peer-reviewed studies of interest and would be more advocacy based (i.e., social media), which would be difficult to analyze.

Several inclusion and exclusion criteria were used to account for educational contexts and settings, such as restricting the study period to the first 2 years of the COVID-19 pandemic and focusing on post-secondary students as the target population. In this study, post-secondary students refer to those pursuing any programs in higher educational institutions, such as bachelor’s and graduate programs in universities, as well as students in technical/community colleges. The term ‘post-secondary’ can capture our target population as studies may merge undergraduate and graduate students together. Given that educational challenges and barriers for post-secondary students differ globally, we focused on studies conducted in OECD countries to investigate inequities among high-income countries with similar educational systems. While the COVID-19 outbreak initiated in December 2019, the wider effect on post-secondary students initiated in March 2020 when the WHO declared COVID-19 a pandemic.^12^ Therefore, it was not expected to find relevant literature prior to March 2020 in OECD countries. Only studies from March 2020 to March 2022 were included to account for the duration of the COVID-19 pandemic. A complete, detailed inclusion and exclusion criteria for this review are provided in Table 1.

**Table 1.** Inclusion and Exclusion Criteria.

Criterion	Inclusion	Exclusion
Time period	March 2020 – March 2022	Studies not within this time period
Language	English	Non-English language
Type of article	Peer reviewed articles	Non-peer-reviewed articles
Study topic	Public health challenges of post-secondary students in higher education during COVID-19	Other education settings
		Other types of students
		Other challenges that do not have public health relevance
Population	Post-secondary students enrolled in a recognized higher education institution (i.e. bachelor’s, graduate, and other degrees in universities, associate’s degrees and other certifications in technical/community colleges)	All other types of students who are not enrolled in higher education
Country of study	OECD countries	Non-OECD countries

### Stage 3: Study Selection

**Figure 1.**
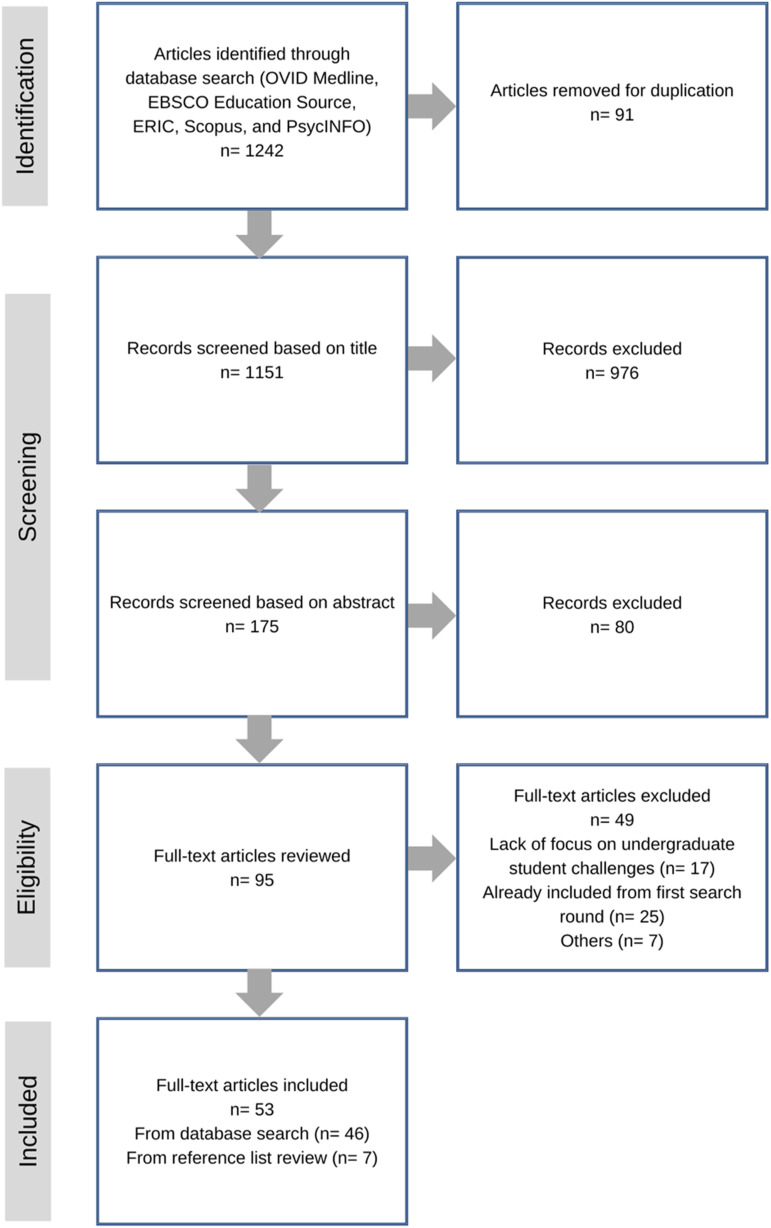
Preferred reporting items for systematic reviews and meta-analyses (PRISMA) flow diagram for study selection.

Using the key search terms, studies were identified for screening in the following order: title, abstract, and full-text. One researcher (PD) independently conducted the title and abstract screening, and was reviewed by the second researcher (LRD). Full-text versions of these articles were obtained and reviewed by one researcher (PD) independently, and consultation with the other author (LRD) was done for any ambiguity or uncertainty. This process of article selection was informed by the Preferred Reporting of Items for Systematic Reviews and Meta-Analyses (PRISMA) statement,^13^ shown in Figure 1.

### Stage 4: Charting Data

Selected studies were charted to document concise summaries of each article. Summaries included the author, year, geographical location, study design, socio-economic variables reported on, including inequities (if any stated in the article), and the public health issue identified from the post-secondary student challenges. One researcher (PD) independently charted all articles and reviewed them with the other author (LRD) to guide the process and resolve any uncertainties.

### Stage 5: Collating, Summarizing, and Reporting the Results

The findings of this scoping review are presented using a narrative synthesis approach. Themes were created and organized based on the type of public health challenge described in the post-secondary population and the socio-economic variables reported in the studies. Self-reported experiences regarding social disadvantage and inequities were also documented within each of the challenges.

## Results

Our initial search across databases examining public health challenges for post-secondary students during COVID-19 in OECD countries yielded 1242 articles, of which 91 duplicates were removed (*n* = 1151). Out of the 1151 article titles screened, 976 were excluded (*n* = 175). The main reasons for the exclusion of article titles were: study setting outside of OECD countries or non-educational settings (i.e., health services), and non-post-secondary student populations. Another 80 articles were excluded based on abstract descriptions because the focus was not related to student learning, or the articles were not original research studies (*n* = 95). Finally, 49 full-text articles were excluded as they were not relevant to the higher education context during the COVID-19 pandemic, or they were already included in the first search round (*n* = 46). After careful review of the reference lists of the included articles, an additional seven articles were added, bringing the total to 53 full-text articles included in this scoping review. A visual schematic of our inclusion process is illustrated in Figure 1 and the analysis of included articles are shown in Table 2.

**Table 2.** Summarized Characteristics of Included Studies.

Characteristic	Studies
Country	USA (*n* = 39)^14–52^
	France (*n* = 4)^53–56^
	Poland (*n* = 3)^57–59^
	Australia (*n* = 2)^60,61^
	Israel (*n* = 1)^62^
	Canada (*n* = 1)^63^
	Germany (*n* = 1)^64^
	Spain (*n* = 1)^65^
	Switzerland (*n* = 1)^66^
Year	2021 (*n* = 39)^14,15,17,18,20–23,25–31,33–36,38,39,41,44–48,50,51,53,54,56,58,60–65^
	2020 (*n* = 12)^16,19,24,40,42,43,52,55,57,59,66^
	2022 (*n* = 2)^37,49^
Type of study	Quantitative (*n* = 39)^14–17,19,22–27,29,30,32,33,36–39,41,42,44,46–48,50,52–59,61,63–66^
	Qualitative (*n* = 6)^21,34,45,51,60,62^
	Mixed-methods (*n* = 8)^18,20,28,31,35,40,43,49^
Use of validated scales	(*n* = 37)^16–18,20,22–24,26–33,36–39,41–44,46–48,50,53,54,56–59,61,63,65,66^

Most articles were based in the USA (39/53),^14–52^ while the rest of the studies were from France (4/53),^53–56^ Poland, (3/53),^57–59^ Australia (2/53),^60,61^ and one study from each of the following countries: Israel,^62^ Canada,^63^ Germany,^64^ Spain,^65^ and Switzerland.^66^ Most studies were published in 2021 (39/53),^14,15,17,18,20–23,25–31,33–36,38,39,41,44–48,50,51,53,54, 56,58,60–65^ compared to 2020 (12/53),^16,19,24,32,40,42,43,52,55, 57,59,66^ and 2022 (2/53).^37,49^ Thirty-nine studies used solely quantitative methods (i.e. online surveys, computerized application-based data collection),^14–17,19,22–27,29,30,32,33, 36–39,41,42,44,46–48,50,52–59,61,63–66^ eight studies used mixed-methods,^18,20,28,31,35,40,43,49^ and six studies utilized only qualitative methods (i.e. open-ended survey questions, in-depth interviews, semi-structured interviews).^21,34,45,51,60,62^ A total of 37 studies used validated measures and scales in their quantitative survey methods.^16–18,20,22–24,26–33,36–39,41–44,46–48,50,53,54, 56–59,61,63,65,66^ The results above are summarized in Table 3. Two studies included university undergraduate and college undergraduates, ^31,33^ while only one study focused on community college undergraduate students.^35^ All 53 studies included undergraduate students (university and college), with 27 studies including university graduate students as well.^14,15,17,18,21,26–29,31–33,37–42,47–49,51,59,61,62,64,65^ Among the 39 US-based studies, 26 studies stated their sample of post-secondary students to be from public universities,^15,17,22,24–30,32,34,36–38,40,42,43,45–52^ 6 studies indicated their sample to be from a mix of public and private universities,^16,18, 21,23,39,41^ and 4 studies stated their study sample to be from private universities.^14,19,20,44^ Of the six USA studies that stated their post-secondary institution study setting serves minority/underserved students,^27,28,32,38,49,51^ five of these studies took place in Hispanic Serving Institutions (HSIs).^27,28,32,49,51^ To our knowledge, no study mentioned the inclusion of Historically Black Colleges and Universities (HBCUs). The most common validated scales used to measure depression and anxiety were The *Patient Health Questionnaire – 8* (PHQ-8) and the *General Anxiety Disorder-7* (GAD-7). Validated scales for perceived stress and food security were also used, particularly the *Perceived Stress Scale* (PSS) and the *US Food Security Survey Module* (US FSSM). Only two validated metrics were used to measure levels of physical activity, namely the *International Physical Activity Questionnaire- Short Form* (IPAQ-SF) and the *Global and Physical Activity Questionnaire*.

**Table 3.** Thematic Analysis of Included Studies.

Authors	Study methods/Design	Findings related to socio-economic variables						Public health issues identified according to thematic analysis						
		Race/ethnicity	Sexual and gender minority	Gender	Income	Rural location	Immigration status	Mental health	Financial insecurity	Physical health	Food insecurity	Social well-being	Digital access	Housing or relocation
*USA Studies*														
^14^	Quantitative online survey via email (*n* = 654)													
^15^	Online cross-sectional questionnaire from 7 universities (*n* = 2534)													
^26^	Pre and post quantitative questionnaire (*n* = 579)													
^37^	Quantitative survey (*n* = 698)													
^47^	Quantitative online surveys via email (*n* = 1434)													
^48^	Quantitative online survey (*n* = 1743)													
^49^	Qualitative and quantitative data via online survey (*n* = 220)													
^50^	Two online quantitative surveys (longitudinal data) (*n* = 419)													
^51^	Online open-ended qualitative survey (*n* = 206)													
^52^	Online quantitative survey administered via professors in university courses (*n* = 39)													
^17^	Online cross-sectional survey (*n* = 2018)													
^18^	Quantitative and qualitative online surveys for college students across the USA (baseline and post survey) (*n* = 707)													
^19^	Quantitative data collected via app, longitudinal study (*n* = 217)	Not described												
^20^	Qualitative semi-structured interviews (*n* = 18) and quantitative online survey (*n* = 312)	Not described												
^21^	Semi-structured interviews (*n* = 12)													
^22^	Online cross-sectional survey (*n* = 1225)													
^23^	Online quantitative survey via 30 universities (*n* = 2913)													
^25^	Quantitative survey (*n* = 1001)	Not described												
^27^	Online cross-sectional survey (*n* = 909)													
^28^	Online qualitative and quantitative surveys and in-depth interviews (*n* = 18)													
^29^	Online cross-sectional survey (*n* = 3206)													
^30^	Online quantitative survey (*n* = 550)													
^38^	Online quantitative survey (*n* = 1821)													
^43^	Interview surveys (quantitative and qualitative data) (*n* = 195)	Not described												
^31^	Online qualitative and quantitative survey (*n* = 228)													
^32^	Online quantitative survey (*n* = 651)													
^33^	Quantitative survey; longitudinal cohort study (*n* = 1647)													
^34^	Online focus groups (*n* = 30)	Not described												
^35^	Online quantitative and qualitative survey (*n* = 356)	Not described												
^36^	Online quantitative survey (*n* = 989)													
^39^	Online quantitative survey (*n* = 509)													
^40^	Online quantitative and qualitative survey data (*n* = 64)													
^41^	Online quantitative survey (*n* = 403)	Not described												
^42^	Online cross-sectional survey before and during COVID-19 (*n* = 2039)													
^44^	Online quantitative questionnaire (*n* = 403)													
^45^	Qualitative interviews (*n* = 34)													
^24^	Cross-sectional survey (*n* = 162)													
^16^	Online quantitative survey (*n* = 477)													
^46^	Online quantitative survey (*n* = 1019)													
*France Studies*														
^53^	Quantitative online survey (*n* = 3764)													
^54^	Quantitative online survey (*n* = 291)	Not described												
^55^	Online quantitative survey (*n* = 291)	Not described												
^56^	Quantitative online survey via email (*n* = 8004)													
*Poland Studies*														
^58^	Online cross-sectional survey (*n* = 2172)													
^57^	Repeated cross-sectional study (*n* = 7228)													
^59^	Online quantitative survey (*n* = 914)													
*Australia Studies*														
^60^	Qualitative interviews (*n* = 27)													
^61^	Online cross-sectional survey (*n* = 787)													
*Israel Studies*														
^62^	Qualitative online, open-ended, questionnaire (*n* = 154)													
*Canada Studies*														
^63^	Longitudinal online surveys (*n* = 510)													
*Germany Studies*														
^64^	Quantitative survey (*n* = 1475)	Not described												
*Spain Studies*														
^65^	Quantitative survey (*n* = 932)													
*Switzerland Studies*														
^66^	Longitudinal data (*n* = 212 compared with *n* = 54)													

### Public Health Challenges of Post-Secondary Students during COVID-19

Various public health issues impacting student learning and achievement were reported in the included articles. In the charted data, we categorized post-secondary public health challenges and any sociodemographic disparities reported by students to analyze the potential public health impact. The six categories included: food insecurity, financial instability, digital access, housing or relocation, mental health and social well-being, and physical health.

#### Food Insecurity

Out of the 53 articles yielded in the search, 12 (23%) articles discussed post-secondary student experiences with food insecurity.^17,21,26,28,29,32,34,38,41,42,47,48^ The prevalence of food insecurity in post-secondary student populations ranged from 17% to 59.6%,^17,29,41,42,47,48^ while the prevalence of risk for food insecurity was found to be high as 65.5% in one study in the USA.^32^ In contrast, one US-based study found that the prevalence of food insecurity did not significantly increase with the onset of COVID-19.^26^ Six studies reported that low socioeconomic groups such as non-white and international students were more likely to be food insecure^17,26,32,42,47,48^ and two US-based studies reported that COVID-19 was worsening food insecurity in socio-economically disadvantaged student populations.^21,28^ Three studies found that student employment loss was associated with increased food insecurity.^17,47,48^ Two other studies that are US-based reported that students who moved to an off-campus location because of COVID-19 were more likely to be food insecure potentially due to a lack of time to cook and a lack of access to campus food pantries.^29,47^ Experiences of financial issues were reported to increase food insecurity among students in two US-based studies.^42,47^ In two US-based articles, living on campus or living alone increased the likelihood of students experiencing food insecurity.^47,48^ One US-based study found that low social support, in the absence of family, friends, and community, may also be a contributing factor to student food insecurity.^28^ Additionally, one Canadian study reported that food-insecure students did not have greater mental health disruptions than non-food-insecure students.^63^

#### Financial Instability

In 25 articles (47%), students reported financial instability during COVID-19^14,15,17,18,21,24,27,29–31, 33,36,38,42,44,45,48,52,55,56,58,60,61,63,66^ with 18 describing the negative impact of the COVID-19 pandemic on financial status and employment for students.^14,17,18,21,27,29,31, 36,38,42,44,45,48,52,55,60,61,66^ Furthermore, eight articles reported financial status or employment changes of the student^15,18,24,30,31,56,58,60^ while one US-based article reported financial/employment changes based on family income as factors for increased stress or depression.^38^ Financial inequities in low sociodemographic groups were described in eight studies where rural,^60^ non-white,^27,36,38,44,52^ women,^31,36^ sexual gender minority,^33^ and first-generation students^52^ experienced disproportionate financial hardship compared to their counterparts. Financial concerns were reported to negatively impact access to resources, which disrupted student learning experiences in four studies.^23,33,52,61^ Only one study that was Canada-based stated that students with low income did not report greater mental health disruptions than those who were of higher income.^63^

#### Digital Access

A total of eight (15%) articles described challenges regarding digital access to online learning.^23,35,49,51,52,62,64^ Students detailed poor internet connectivity, technological issues, and limited access to digital devices as factors for online learning difficulties.^23,35,49,51,52,62^ A study in Germany found that limited digital access due to financial constraints was a barrier to online learning as well.^64^ One US-based study suggested sociodemographic inequities in digital access were due to skill deficiency and lack of computer resources.^51^ Another study from the USA highlighted students reporting concerns with the digital divide, accessibility, and exacerbated inequities during the COVID-19 online learning experience.^40^

#### Housing or Relocation

Only six (11%) articles reported housing or relocation difficulties among students during the COVID-19 pandemic.^15,47,52,55,60,63^ Of these studies, three articles found that students living on campus, living on their own, or experiencing difficulties finding housing, reported an increase in stress.^15,47,55^ Additionally, students who lived alone, lived on campus, or had difficulty finding housing, also worked increased hours to support themselves financially during the pandemic, based on one USA study.^47^ In contrast, one Canada-based study found that students who moved back home with their families or to another location did not report greater mental health disruptions than students who did not move from campus or an off-campus location.^63^ Another study based in the USA serving students in a course showed that housing insecurity was experienced by 15% of students.^52^ One Australian study depicted the experiences of rural students feeling stress from choosing between staying on campus and relocating, as staying on campus came with social isolation while relocating required financial resources and practical support.^60^

#### Mental Health and Social Well-Being

In total, 66% (*n* = 35) of included studies suggested the pandemic impacted student mental health and well-being.^14–16,18,19,22,24,25,27,30,31,33, 35–40,43–46,50,52–59,61,63,65,66^ Out of these 35 articles, 27 reported students experiencing increased stress, anxiety, and depression, or had symptoms of anxiety and depression during the COVID-19 pandemic.^14,15,18,19,24,25,27,31,33,35–37, 40,43,45,46,50,53–59,63,65,66^ Furthermore, 24 studies described sociodemographic inequities in mental health outcomes with women,^15,18,24,30,37,40,46,50,52,53,56–59,61,65,66^ racialized, and first-generation, ^15,18,22,44,50,52^ low-income,^15,18,30,38,61^ and sexual gender minority students^16,18,36,39,50^ being the most vulnerable. Two US-based studies reported sexual gender minority, female, and non-white students experiencing barriers to accessing medical care during COVID-19.^36,52^ Additionally, one US-based study discovered that more than 20% of students were unable to access mental health services.^27^ Only three studies compared mental health status before and during the pandemic, demonstrating that rates of anxiety and depression increased.^19,33,50^ A total of 15% (*n* = 8) of studies reported on social well-being and isolation.^14,16,22,31,43,50,62,66^ Of these eight studies, six of them found that students experienced increased stress, anxiety, and depression due to a lack of social support from friends and family and a lack of social connectedness.^14,31,43,50,62,66^ Only three studies reported social isolation and social support difficulties across sociodemographic groups.^16,22,31^

#### Physical Health

There were 13 (25%) studies that investigated changes in physical health and health behaviours such as sedentary behaviours, sleep duration, and alcohol and substance use.^14,15,19,20,25,30,39–41,43,46,52,53^ Five studies found that physical activity decreased, and sedentary behaviours increased among students since the onset of the pandemic.^14,19,30,41,46^ Sleep was also found to be negatively impacted during the COVID-19 pandemic in four studies.^15,30,43,52^ In another four studies, there was a reported increase in alcohol consumption,^20,25,41,53^ as well as decreased alcohol consumption during the pandemic in one study based in the USA.^20^ Additionally, one USA-based study found increased alcohol use among sexual and gender minority students.^39^ Increased tobacco use among students was described in one France-based study.^53^

## Discussion

We reviewed the literature about public health challenges experienced by post-secondary students in OECD countries during the COVID-19 pandemic. The key self-reported challenges experienced by post-secondary students related to the following six themes: food security, financial instability, digital access, housing or relocation, mental health and social well-being, and physical health. These challenges appear to be pronounced in students from low sociodemographic populations based on self-report, highlighting a pronounced need for solutions that intersect public health and pedagogical interventions. The following discussion examines these challenges related to the social determinants of health.^3^

### Food Insecurity

We found in our study that during the pandemic, students reported food insecurity as having a key impact on their health and well-being.^17,29,32,41,42,47,48^ Only USA studies in our review reported food insecurity metrics and prevalence measures that ranged widely across studies (17% to 59.6%), with the risk of food insecurity being 65.5% in one study with a sample size of 651 post-secondary students.^32^ This is consistent with literature from the USA prior to the pandemic reporting high food insecurity among students.^67-69^ However, among the USA studies included in our review that did not quantify prevalence, the studies emphasized the worsening food security status as a consequence of the pandemic.^21,28,34,38^ According to *The State of Food Insecurity and Nutrition in the World* report, the prevalence of global undernourishment increased from 8.4% in 2019 to 9.9% in 2020 during the pandemic across global populations.^70^ The literature also suggests that students who remain on campus or live alone may experience increased food insecurity as there is a lack of social support, such as family and friends, who often aid in providing food and financial assistance.^71^ Travel restrictions prohibited returning home for some students, while others opted to remain away from home, anticipating an eventual return to their home country or region^72^; these living arrangements could potentially explain higher food insecurity among these students because of isolation and closures of campus food retailers, creating additional barriers to accessing food.^73^

One longitudinal study included in our review with a sample size of 579 reported that COVID-19 did not significantly increase food insecurity.^26^ This is contrary to the literature that suggests that for post-secondary students, COVID-19 has caused employment cuts and housing instability,^74^ which increases the likelihood of being food insecure.^75-77^ However, the authors emphasized that the students in the sample already had a high baseline prevalence of food insecurity prior to the pandemic, making it difficult to detect statistically significant changes during the pandemic.^26^ Research shows that increased food insecurity is associated with increased depressive symptoms.^67,68,78^ Our study findings are consistent with this trend showing worsening mental health status for food-insecure students reported across several studies. However, a Canadian study included in our review by Howard et al. with a sample size of 510 reported that food-insecure students in a Canadian university did not experience greater mental health disruptions than non-food-insecure students,^63^ potentially due to students being eligible to receive federal financial assistance (Canadian Emergency Student Benefit (CESB)),^79^ which could have helped mitigate negative health and social outcomes. However, the sustainability of these programs is unclear and may not be possible in other countries. Additionally, these financial stimuli are temporary and only superficially address the systemic challenges of food insecurity for those at risk for or experiencing food insecurity.

Additionally, the pandemic has exacerbated food insecurity in low socioeconomic groups, namely those who identify as non-white and first-generation students,^74,80^ which is consistent with pre-pandemic trends.^68,78,81^ Campus food deserts and barriers to accessing affordable, nutritious food options predate the pandemic^82^ thus, the collective evidence of the studies in this review emphasizes an urgent call to action. Overall, addressing food insecurity for post-secondary students is consequential, as it is well known that compromised psychosocial health impedes academic achievement,^67^ which in turn can impact the longitudinal trajectory of future health outcomes.^83^

### Financial Instability

This study also revealed that financial precarity intensified alongside reduced employment opportunities for students during the pandemic.^74,80,84^ Increased financial and employment changes were risk factors for stress and depression, which is reflected in research prior to the pandemic as well.^85–88^ Studies undertaken pre-pandemic are consistent with our finding that greater financial difficulties are associated with poorer mental health, and those identifying as women, and non-white ethnicity.^88^ Low socioeconomic groups have worse social and economic conditions that increase their susceptibility to poorer health outcomes^3^ thus, it is not surprising that these groups were disproportionately impacted by the pandemic.^89^ Lack of affordability for essential educational resources, such as course textbooks and technology, can disrupt online learning experiences,^90^ which was reflected in our study findings.^64^ As financial burdens can impede the ability to meet basic needs and deteriorate social and psychological well-being, collectively these social determinants contribute to academic disruption for students.^87^ In fact, higher dropout rates from postsecondary education have longitudinal consequences, exacerbating generational cycles of low socioeconomic status.^75^

### Digital Access

Few studies in our review have reported on challenges with digital connectivity for students, including barriers such as lack of digital access to technology, financial precarity, and lack of technological infrastructure.^90^ With increasing reliance on online learning during the pandemic, the digital divide has been a significant barrier to equitable access to higher education,^91^ which has been shown to impact opportunities and future health outcomes.^92^ As our study findings suggest, lower socioeconomic groups encountered decreased digital access, consistent with the literature indicating that the pandemic exacerbated this existing digital divide in higher education.^91^ Many students experiencing low socioeconomic conditions rely on computers and free internet on campus.^91^ However, with university closures, these students are especially vulnerable to falling behind during online learning.^80,93^ In addition, students living in rural settings are a high-risk group for this trend as they often have low access to broadband internet,^91^ as identified in our study. Most students in the studies we reviewed reported having one digital device to access their online learning, yet internet and technological issues were still prevalent due to many household members using the internet, which contributed to slower internet.^35^ The contemporary challenges of the digital divide have therefore shifted from solely digital ownership to digital sustainability and maintenance, especially in countries where device availability is widespread, like the USA.^94^ Pedagogical approaches that consider innovative ways to achieve equitable solutions to this challenge are essential to bridge gaps, while global and regional policy-level interventions are required to address the need for sustainable structural digital access.

### Housing or Relocation

A few studies have reported on student challenges around housing and relocation in our review. Housing insecurity in the post-secondary student population is not a new phenomenon; just before the pandemic in the USA, 52.3% of students in 22 postsecondary institutions were found to be housing insecure.^75^ Similarly, in the studies we reviewed, students who experienced housing insecurity during the pandemic also experienced increased stress, depression, and anxiety, which is reported in prior literature as well.^75,76,95^ In the USA, with the closure of campuses due to COVID-19 measures, it is anticipated that housing insecurity has increased due to students having to find housing abruptly during COVID-19.^80^ Based on trends leading up to the pandemic, housing insecurity would likely disproportionately impact first-generation,^76^ low-income,^75^ woman-identifying,^77^ and students of colour.^96^ This can potentially be explained by how the price of college and living expenses in countries like the USA has increased drastically since 2008,^97^ and this issue is further experienced by historically marginalized groups who are now attending higher education institutions in ever-increasing numbers.^96^ Many students with lower socioeconomic status manage multiple work and family obligations, such that they may forego their basic needs, including stable housing, in pursuit of higher educational goals.^76^ Additionally, increased housing insecurity has been found to be associated with lower GPA in multiple studies.^75,95,96^; increased housing insecurity can contribute to a higher incidence of mental and physical health challenges as found in other populations.^98^

### Mental Health and Social Wellbeing

Post-secondary students are considered a vulnerable population for potential mental health disorders, particularly anxiety and depression.^80,99^ With COVID-19 stressors being an additional burden for this population, it is expected that mental health has deteriorated for post-secondary students during the pandemic.^80,84,100,101^ Multiple studies in this review measured mental health indicators such as stress, depression, and anxiety; all of which were reported by students to be increasing during the COVID-19 pandemic. In addition, the literature suggests that students with lower socioeconomic status are likely to be disproportionately impacted by worse mental health outcomes,^80,84,102^ which is consistent with our study findings showing mental health inequities for woman-identifying, racialized, first-generation, low-income, and sexual and gender minority students. The closure of educational institutions has impacted daily routines, social supports, access to resources including food and health services, social interactions that maintain good mental health,^84^ and even reprieve from unsafe living conditions.^80^ As schools often offer a primary access point to mental health services and referrals for children and adolescents, the closure of educational institutions could mean more mental health issues are going unidentified,^84^ highlighting a downstream consequence of the increased burden of mental health. Many students in higher education will also access these mental services through their institutions, and therefore this group may also be vulnerable to the effects of school closures.^101^

### Physical Health

While most studies in our review investigated the mental health impacts from the COVID-19 pandemic, some studies also reported on changes in physical health and health behaviours. Greater perceived stress during the pandemic, including social isolation,^103^ was associated with excess alcohol consumption.^104^ Research from Canada indicates increasing alcohol consumption for young people aged 18-34 years since social distancing measures were put in place, with Black, Indigenous, and People of Colour (BIPOC) disproportionately engaging in higher-risk substance use.^102^ We also found studies in our review that showed students reporting shortened or disrupted sleep during the pandemic could potentially be explained by greater screen time,^104^ going to bed at different times, and elevated stress and anxiety levels.^105^ Increased sedentary behaviours and decreased physical activity were also observed during the pandemic, as reported in our study, due to pandemic lockdowns and restricted spaces that limited the ability to engage in usual forms (places and spaces) of engaging in healthy behaviours and physical activity.^105,106^ The longer-term implications of increased sedentary behaviours on other comorbidities, such as diabetes and obesity, remain to be examined in future studies.

### Existing Interventions to Mitigate Student Challenges

Mitigating food, housing, and financial insecurity have historically been driven by downstream policy interventions in reaction to socioeconomic challenges. These policies tend to be limited in their accessibility for affected populations and in some cases, the sustainability of these programs is unclear. For example, federal food assistance programs, such as the Supplemental Nutritional Assistance Program (SNAP) in the USA, have been criticized for restrictive eligibility criteria that exclude most undergraduate students in need of assistance,^69^ with only a 20% participation rate from the student population compared to an 85% participation rate in the general USA population.^82,107^ In response to the pandemic, the USA government introduced three programs that aimed to improve food and economic security: Emergency Ensuring Access to SNAP (EATS) Act, End Pandemic Hunger for College Students Act,^74^ and the Coronavirus Aid, Relief, and Economic Security (CARES) Act.^108^ However, many college students are ineligible for these because they are considered dependants on their parent’s tax returns.^69^ In contrast, Canada introduced the Canada Emergency Student Benefit (CSEB) for students who were not eligible for the Canada Emergency Recovery Benefit (CERB).^79^ Canada’s federal government also suspended the accumulation of interest on student loans until March 2023^109^ and doubled non-repayable Canada student grants for full-time and part-time students who apply for educational financial assistance programs.^110^ Canada also introduced the Emergency Food Security Fund at the beginning of the pandemic that distributed $100 million to Canadian food banks and national food rescue organizations.^111^ However, given that these programs were in reaction to the priority and urgency of COVID-19, their practical and financial sustainability remains unclear, and their continuation will be subjected to the political will, climate, and priorities of future political parties.

Australian and New Zealand governments have been allocating increasing funding and resources to alleviate digital access issues in higher education.^112^ The USA government introduced the Basic Assistance for Students in College (BASIC) Act to allocate funding to higher education institutions to support students from marginalized communities with technology access.^113^ While these relief efforts are important to support students during emergency learning, technological maintenance continues to be a prominent barrier for post-secondary students outside of emergency learning circumstances.

To support student mental health and substance use addiction during the pandemic, OECD countries have implemented secondary prevention approaches at federal and institutional levels. Australia has “Headspace” centres that support mental health for young people aged 12-25 by partnering with various community organizations.^84^ France has facilitated university students to have three free mental health consultations^84^ while the UK and Canada have dedicated increased funding for community-based helpline and app-based mental health services for youth and young adults.^114,115^ However, poor mental health is exacerbated by the COVID-19 pandemic and the sustainability of these interventions are dependent on COVID-19 priority efforts dictated by legislative bodies. Moreover, poor student mental health is related to underlying systemic barriers that existed before the COVID-19 pandemic.^116^

The sustainability of interventions for food and financial insecurity, poor mental health, and digital access is unclear as they are downstream and reactive to COVID-19. Evidence shows that more sustainable ways to address insecurities, such as increasing living wages, have improved workers’ health and well-being, as well as productivity.^117^ Basic income programs, such as the Canadian Guaranteed Annual Income (GAI) program and Old Age Security (OAS) programs for seniors, have been found to reduce food insecurity,^118^ and have improved mental, physical, and functional well-being.^118^ Principles of universal design can support the development of more inclusive and systemic structural and institutional-level change. By designing effective solutions that support the specific needs of vulnerable subgroups - the long-term positive outcomes can benefit the broader population.^119^

### Strengths and Limitations

This scoping study includes many strengths. First, this study captures a breadth of literature regarding public health challenges for students during COVID-19. We identified six key themes around these public health challenges in the context of several social determinants of health: food insecurity, financial insecurity, digital access, housing insecurity, mental health and social well-being, and physical health. Second, we focused on higher-income nations as described by the membership of the OECD to highlight that notwithstanding the high-income status of a country, inequities are apparent and often overlooked. Third, identifying shortcomings of policy-driven efforts in response to the pandemic can help decision-makers plan for more systemic, upstream approaches to sustainably and equitably address the identified public health challenges. Fourth, quantitative studies included in our review had large sample sizes, allowing for increased generalizability of the current review. Finally, data from qualitative studies included herein were helpful to capture self-described viewpoints directly from students. Despite these strengths, there are some important limitations to our study. First, quality assessment procedures are not included in the scoping review framework hence, we were not able to assess the robustness of results in the studies in this review. Second, we did not report the prevalence rates of most of the student public health challenges, given the lack of congruent analyses between studies and the absence of prevalence rates reported. Overall, our scoping study provided consolidated evidence on post-secondary public health challenges and can offer insight into opportunities for addressing long-term, sustainable solutions for student health and well-being.

## Conclusion

Education is a key social determinant of health and has longitudinal consequences for populations. Therefore, it is critical that in the aftermath of the COVID-19 pandemic, future pandemic preparedness should involve careful consideration of structural and institutional, systems-level support for students. Interventions that address the social determinants of health before the issues worsen and interventions that aim to reduce the incidence of poor health outcomes should be implemented, whereby policy-level changes and partnerships within and external to higher education institutions can be leveraged to meet the needs of student communities. Examples such as increasing eligibility for federal aid programs to include post-secondary students, increasing youth employment and living wages, increasing funding for culturally safe and accessible mental health services, and increasing affordable student housing, may each be opportunities to reimagine the way we think about student health and well-being. Educational institutions might also benefit from leveraging student support for online learning infrastructures and alleviating online learning pressures through pedagogical and policy-level changes that support innovation, growth, and social responsibility – involving students as partners in the discovery and evaluation of solutions. This scoping review provides an overall understanding of the key challenges faced by post-secondary students in high-income countries during the COVID-19 pandemic, framing these challenges as social determinants of health, which can guide future research and decision-making for student health and well-being in a post-pandemic era.

## Supplemental Material

Supplemental Material - Public Health Challenges for Post-secondary Students During COVID-19: A Scoping Review Supplemental Material for Public Health Challenges for Post-secondary Students During COVID-19: A Scoping Review by Pooja Dey, Leanne Kenney De Souza in Community Health Equity Research & Policy
