# Supplementary material for: Public Health Challenges for Post-secondary Students During COVID-19: A Scoping Review
Source: Community Health Equity Res Policy. 2024 May 31;45(4):429–46. doi: 10.1177/2752535X241257561 (PMC12059239; doi:10.1177/2752535X241257561)
Supplement: Supplemental Material - Public Health Challenges for Post-secondary Students During COVID-19: A Scoping Review [file sj-pdf-1-qch-10.1177_2752535X241257561.pdf]

Search strategy

On November 21st, 2021, the first round of the search strategy was conducted and the second round search was conducted on March 9th, 2022, both done by PD. Both searches were limited from March 2020 to March 2022. The following tables shows the total results from both search rounds and PRISMA selection of articles.

Round 1 search November 21st, 2021

Database	Search results
OVID Medline	65
ERIC	74
PsycINFO	36
Scopus	685
EBSCO Education Source	11
Total	871

PRISMA Selection from round 1 search

Records found	871	52 duplicates removed
Records screened based on title	819	719 excluded
Records screened based on abstract	100	46 excluded
Full-text screening	54	15 excluded
Full-text included	39	
Full-text added from reference lists	7	
Total full-text included	46	

Round 2 search March 9th, 2022

Database	Search results
OVID Medline	77
ERIC	92
PsycINFO	44
Scopus	122
EBSCO Education Source	36
Total	371

PRISMA Selection from round 2 search

Records found	371	39 duplicates removed
Records screened based on title	332	257 excluded
Records screened based on abstract	75	34 excluded
Full-text screening	41	34 excluded
Full-text included	7	
Full-text added from reference lists	0	
Total full-text included	7	

OVID Medline

Ovid MEDLINE: Epub Ahead of Print, In-Process & Other Non-Indexed Citations, Ovid MEDLINE® Daily and Ovid MEDLINE® <1946-Present>

1 exp COVID-19/
2 exp Universities/
3 exp Health Status Disparities/ or exp Social Class/ or inequality.mp. or exp Income/ or exp Socioeconomic Factors/
4 1 and 2 and 3
5 limit 4 to yr="2020 -Current" (Round 1, Round 2: 65, 77)

ERIC

Applied to all: Limit to peer-reviewed and publications March 2020 to present

ti("post-secondary" OR universit* OR college OR "higher ed*" OR pedagogy OR "distance ed*" OR "online learning") AND (COVID-19 OR coronavirus OR SARS-CoV-2) AND (inequalit* or unequal or ((ethnic or socioeconomic) N2 (disparit* or inequit*)) OR "digital divide" OR "digital inequalit*")
Round 1, Round 2: (54, 70)

ti("post-secondary" OR universit* OR college OR "higher education" OR pedagogy OR "distance education" OR "online learning") AND (internet access OR (comput* OR cellular OR mobile OR phone OR technolog* OR smartphone* OR headphone* OR earphone* OR tablet) N2 access*) AND (COVID-19 OR coronavirus)
Round 1, Round 2: (10, 14)

ti("post-secondary" OR universit* OR college OR "higher ed*" OR pedagogy OR "distance ed*" OR "online learning") AND (“financial stress*” or unemploy* or “job insecurit*” OR “job loss” OR “ food insecurit*” OR “housing insecurit*”) AND (COVID-19 OR coronavirus OR SARS-CoV-2)
Round 1, Round 2: (10, 8)

PsycINFO

Applied to all: Limit to peer-reviewed and publications March 2020 to present

ti("post-secondary" OR universit* OR college OR "higher ed*" OR pedagogy OR "distance ed*" OR "online learning") AND (COVID-19 OR coronavirus OR SARS-CoV-2) AND (inequalit* or unequal or ((ethnic or socioeconomic) N2 (disparit* or inequit*)) OR "digital divide" OR "digital inequalit*")
Round 1, Round 2: (27, 36)

ti("post-secondary" OR universit* OR college OR "higher education" OR pedagogy OR "distance education" OR "online learning") AND (internet access OR (comput* OR cellular OR mobile OR phone OR technolog* OR smartphone* OR headphone* OR earphone* OR tablet) N2 access*) AND (COVID-19 OR coronavirus)
Round 1, Round 2: (6, 4)

ti("post-secondary" OR universit* OR college OR "higher ed*" OR pedagogy OR "distance ed*" OR "online learning") AND ("financial stress*" or unemploy* or "job insecurit*" OR "job loss" OR " food insecurit*" OR "housing insecurit*") AND (COVID-19 OR coronavirus OR SARS-CoV-2)
Round 1, Round 2: (3, 4)

Scopus

Applied to all: Search within article title, abstract, keywords
Applied to all: Published from 2020 to present

"coronavirus 2019" or COVID-19 or 2019-nCoV or SARS-CoV-2 or "severe acute respiratory syndrome coronavirus 2" or "novel coronavirus" or "nCoV disease" or SARS2 or COVID19 or 2019nCoV or "coronavirus disease-19" or "coronavirus disease" or "2019 novel coronavirus"
AND
"post-secondary" or universit* or college or "higher ed*" or pedagogy
AND
"distance ed*" OR "online learning" OR "e-learning" OR "distance learning"
AND
"internet access*" OR "access to device*" OR "access to computer*" OR "digital divide" OR "digital inequalit*" OR "digital inequit*"
OR inequal* OR inaccessib* OR inequit* OR "ethnic disparit*" OR "socioeconomic disparit*" OR disparit* OR "job insecurit*" OR unemploy* OR "employment insecurit*" OR "financial stress*" OR "food insecurit*" OR "housing insecurit*"
Round 1, Round 2: (685, 122)

EBSCO Education Source

Applied to all: Search modes: Boolean/phrase
Applied to all: Expanders: Apply equivalent subjects
Applied to all: Limiters: Published date: 20200101-20221231

KW(post-secondary or universit* or college or higher ed* or pedagogy) or TI(post-secondary or universit* or college or higher ed* or pedagogy)

KW(covid-19 or coronavirus or 2019-ncov or sars-cov-2 or cov-19) or TI(covid-19 or coronavirus or 2019-ncov or sars-cov-2 or cov-19)

KW(online learning or e-learning or distance learning or remote learning or distance ed*) or TI(online learning or e-learning or distance learning or remote learning or distance ed*)

digital N3 (inequalit* or divide)

KW access* N3 (device or internet or web* or phone or comput* or mobile or technolog* or smartphone or headphone* or earphone*)

S4 or S5

KW (inequalit* or unequal or ((ethnic or socioeconomic) N3 (disparit* or inequit*)))

KW((financ* or employ* or hous* or food) N3 (stress* or insecurit*)) or unemploy*

S1 AND S2 AND S3 AND S6 (Round 1, Round 2: 8, 22)

S1 AND S2 AND S3 AND S7 (Round 1, Round 2: 3, 6)

S1 AND S2 AND S3 AND S8 (Round 1, Round 2: 0, 8)
